# Pathogenic Potential and Control of *Chryseobacterium* Species from Clinical, Fish, Food and Environmental Sources

**DOI:** 10.3390/microorganisms10050895

**Published:** 2022-04-25

**Authors:** Elebert Pauline Mwanza, Arno Hugo, George Charimba, Celia J. Hugo

**Affiliations:** 1 Department of Microbiology and Biochemistry, University of the Free State, P.O. Box 339, Bloemfontein 9300, South Africa; elebertweb1@gmail.com; 2 Ministry of Health, Food and Drugs Control Laboratory, Lusaka 10100, Zambia; 3 Department of Animal Science, University of the Free State, P.O. Box 339, Bloemfontein 9300, South Africa; hugoa@ufs.ac.za; 4 Department of Food Science and Technology, Cape Peninsula University of Technology, Bellville 7535, South Africa; charimbag@cput.ac.za

**Keywords:** *Chryseobacterium*, pathogenic, enzymes, antimicrobial, disinfectant, resistance

## Abstract

*Chryseobacterium* species are isolated and taxonomically evaluated from a wide range of sources. While *C. gleum* and *C. indologenes* have been implicated in human disease, the potential pathogenicity of numerous other species have not been investigated. The aims were therefore to evaluate 37 *Chryseobacterium* species and *Elizabethkingia meningoseptica* from environmental, food, fish, water and clinical sources for production of haemolysis, growth at 37 °C, and production of virulence enzymes. The control of these strains were investigated by determination of antimicrobial and disinfectant resistance. All the species produced α- or β-haemolysis. In terms of growth at 37 °C and production of virulence enzymes, *C. soldanellicola* (environmental), *C. oranimense* (food) and *C. koreense* (natural mineral water) could be potential human pathogens. *Chryseobacterium piscium* might be pathogenic to fish. Trimethoprim could be the most effective antimicrobial for the treatment of a *Chryseobacterium* species infection, while the disinfectants that contain poly-dimethyl ammonium chloride or benzalkonium chloride could be regarded as the most effective for decontamination of surfaces contaminated with *Chryseobacterium* species.

## 1. Introduction

*Chryseobacterium* is a genus that is evolving rapidly and currently consists of 120 validly published species [1]. It belonged to the family *Flavobacteriacea* [2] until recently, when it was allocated into the novel family *Weeksellaceae* [3].

The species of *Chryseobacterium* occurs widely in environmental, food, and water sources, and some have been isolated from the clinical environment, humans, and animals, with others that have been implicated in causing disease in fish and humans [4]. In food, they are generally regarded as spoilage bacteria because most are psychrotolerant and produce proteolytic enzymes [5,6,7], while some produce biogenic amines [8]. Many species of *Chryseobacterium* are also known to be resistant to several antimicrobials [9].

However, apart from antibiotic resistance, the pathogenicity of most of the *Chryseobacterium* species has not been well studied. There are different pathogenic characteristics that may be used to assess the pathogenic potential of microorganisms. These include screening for haemolytic activity, determining the virulence enzymes that the organism produces [10,11] and checking for its resistance/sensitivity to antimicrobials.

To the authors’ best knowledge, no study yet has investigated ways in controlling the growth of *Chryseobacterium* species isolated from environmental and food sources. Hence, methods of eliminating them from surfaces and/or utensils and/or wounds should be understood and correctly applied to prevent further transmission. Disinfectants may be used as a way of decontamination. However, for the effectiveness of the disinfectant to be enhanced, the correct concentration should be applied; otherwise, a disinfectant that is bactericidal may be converted into a bacteriostatic disinfectant at lower concentrations [12].

The aims of this study were therefore to determine the virulence characteristics of *Chryseobacterium* species from food and environmental sources and to compare their characteristics to *Chryseobacterium* species that are pathogenic to humans and fish by assessing the ability to haemolyse blood cells, produce virulence enzymes and to being resistant to antimicrobials and disinfectants. To the authors’ knowledge, this is the first study performed on the pathogenic potential of food, water and environmentally isolated members of the genus *Chryseobacterium*.

## 2. Materials and Methods

### 2.1. Strains Used and Resuscitation of Cultures

All the strains used were type strains obtained from culture collections as indicated in Table 1, and they were maintained in a freeze-dried form. The strains were chosen on the basis of being isolated from food, water and the environment. Human pathogenic (*C. gleum*, *C. indologenes*) and fish pathogenic (*C. balustinum*, *C. scophthalmum*) strains were included to act as reference strains for the determination of the pathogenic characteristics in this study. *Elizabethkingia meningoseptica* was also included since it was formerly associated with the genus as (*C. meningosepticum*) and is a human pathogen [13].

The strains were resuscitated in 10 mL nutrient broth (Oxoid CM0001) and incubated at 25 °C for 48 h. Purity of the strains was checked by streaking on nutrient agar (Oxoid CM0003) and by incubating at 25 °C for 48 h. Pure single colonies were streaked on nutrient agar slants which were used as the working cultures after an incubation period of 48 h at 25 °C. The nutrient agar slants were stored at 4 °C. Sub-culturing of the working cultures was performed every 7–8 weeks.

### 2.2. Preparation of Cell Cultures for Determination of Pathogenic Characteristics

For the preparation of cell cultures for determination of haemolysis, growth at 37 °C and enzyme production, the cultures were standardised by streaking from the nutrient agar slants on nutrient agar and incubation at 25 °C for 48 h. Growth from the agar plates was suspended in 9 mL of sterile 0.1 mol/L phosphate buffer until a density comparable to McFarland 1 standard (Difco 0691326). The transmittance was checked (Biolog^TM^, Anatech Instruments, Johannesburg, South Africa) and standardised at 81 ± 3%. For confirmation, serial dilutions (1:10) were also prepared for each of the strains from 10^−1^ to 10^−8^, although plating on nutrient agar was only performed from the 10^−4^ to 10^−8^ dilutions in order to obtain a target population equivalent to 6 log CFU/mL. 

### 2.3. Qualitative and Quantitative Enzyme Production

Ten microlitres of each standardised species suspension was spotted (10 μL) on media containing the substrate to detect the type of haemolysis, growth at 37 °C, or the production of a specific enzyme. Each inoculum was spotted in triplicate on two plates to give a total of six data points. Incubation was at 25 °C for 72 h since all the strains in this study were able to grow at this temperature. Qualitative (present/absent) as well as quantitative (Z-scores) analyses were performed.

Haemolysis production was performed according to Buxton [14] on pre-poured 5% sheep blood agar (Selecta Media 510131). The production of virulence enzymes was performed according to Edberg et al. [10] and Pavlov et al. [11] as follows: protease production on brain heart infusion (Oxoid CM1135) agar containing 3% (*w*/*v*) skim milk powder (Difco); lipase production on trypticase soy agar (Oxoid CM0129) supplemented with 1% (*v*/*v*) Tween 80 (Merck); DNase production on DNase agar (Oxoid CM321) supplemented with 0.1 g/L toluidine blue O; hyaluronidase production on brain–heart infusion medium (Oxoid CM1135) supplemented with 2 mg/mL of hyaluronic acid (Sigma) and 5% (*v*/*v*) bovine albumin fraction V (Sigma); chondroitinase production on brain–heart infusion medium supplemented with 4 mg/mL of chondroitin sulphate A from bovine trachea (Sigma) and 5% (*v*/*v*) bovine albumin fraction V (Sigma); lecithinase production on nutrient agar supplemented with 50% (*v*/*v*) egg yolk emulsion (Oxoid); fibrinolysin production on nutrient agar supplemented with 280 mg/L of fibrinogen type III from human plasma (Sigma F3879); elastase production on nutrient agar with a 1% (*w*/*v*) suspension of elastin from bovine neck ligament (Sigma); gelatinase production was performed according to MacFaddin [15] with some modifications. Nutrient broth (Oxoid CM67; 500 mL) was supplemented with 5.5 g of agar (Oxoid LP0011) and boiled until the agar was completely dissolved. The medium was allowed to cool slightly before 2 g of gelatin (Merck 260 31 00 EM) was added. The medium was allowed to stand for 5 min followed by autoclaving at 121 °C for 5 min. Incubation was for 5 days at 25 °C before being flooded with 5–10 mL of Frazier’s Reagent (12 g mercuric chloride + 80 mL distilled water + 16 mL concentrated HCl). Clear zones around the inoculated test organism were indicative of a positive result (presence of enzyme).

### 2.4. Antimicrobial Resistance/Susceptibility

The antimicrobial resistance patterns of the test organisms were determined using the Kirby–Bauer Disk Diffusion method as prescribed by the Clinical and Laboratory Standards Institute [48]. The different classes of antimicrobials used were cell wall synthesis inhibitors (ampicillin 10 μg, amoxicillin 10 μg, vancomycin 30 μg, cephalothin 30 μg), protein synthesis inhibitors (neomycin 30 μg, tetracycline 30 μg, oxytetracycline 30 μg), a 30 S ribosomal subunit inhibitor (streptomycin 25 μg), a 50 S ribosomal subunit inhibitor (chloramphenicol 30 μg) and a folic acid synthesis inhibitor (trimethoprim 2.5 μg). These antimicrobials were supplied by ThermoFisher (Johannesburg, South Africa).

Bacterial suspensions of each species were prepared with densities equal to a MacFarland 1 standard in phosphate buffer as indicated above. The suspensions were streaked with cotton swabs on two Mueller–Hinton agar (Oxoid CM337) plates per strain. Antimicrobial disks were placed in triplicate onto the inoculated plates, resulting in six data points per strain. The plates were incubated for 48 h at 25 °C. The diameters of the zones of clearance around each disk were measured and analysed according to the performance standards for antimicrobial susceptibility testing using *Enterobacteriaceae* to interpret the results, since *Chryseobacterium* does not have specific criteria in CLSI [48]. The classification of resistance and susceptibility to an antimicrobial was as follows: resistance (R), intermediate (I) and susceptible (S) (all values in mm): ampicillin and amoxicillin (R ≤ 13; I 14–16; S ≥ 17); vancomycin, and cephalothin (R ≤ 14; I 15–17; S ≥ 18); neomycin, tetracycline, oxytetracycline and streptomycin (R ≤ 11; I 12–14; S ≥15); chloramphenicol (R ≤ 12; I 13–17; S ≥ 18) and trimethoprim (R ≤ 10; I 11–15; S ≥ 16).

### 2.5. Disinfectant Resistance/Susceptibility

Fourteen of the 38 strains in this study were chosen for the disinfectant resistance study based on being reference strains that are isolated from all types of sources in this study and based on the virulence characteristics in this study (indicated with an asterisk in Table 1). The four disinfectants tested against the 14 *Chryseobacterium* species are commercially available. The active ingredient of disinfectant 1 was chloroxylenol, disinfectant 2 was benzalkonium chloride, disinfectant 3 was chlorhexidine gluconate (cetrimide) and disinfectant 4 was poly-dimethyl ammonium chloride. Disinfectants 1 and 3 are marketed for use on wounds in diluted form and disinfectants 2 and 4 for the cleaning of surfaces (clinical and food processing).

The Minimum Inhibitory Concentration (MIC) method was used for determination of resistance of the test organisms to disinfectants. A two-fold dilution range was prepared for each disinfectant to be tested. The initial concentration prepared was 1% (*v*/*v*), and the concentration was halved until another four concentrations of 0.5%, 0.25%, 0.125% and 0.0625% were obtained. Into each dilution was added a 100 µL of test organisms from a broth culture which was not older than 24 h. The dilutions were then left for 20 min (contact time). After 20 min, a 100 µL of each dilution was added to 5 mL of nutrient broth and incubated at 25 ºC for 72 h. At the same time, both a positive control (test organism) and a negative control (disinfectant) were prepared and incubated. The last tube of nutrient broth not to show growth was regarded as the minimum inhibitory concentration of the disinfectant for that particular test organism. However, this was the case only if the positive control showed growth while the negative control showed no growth.

### 2.6. Statistical Analysis

The data obtained from the determination of enzyme activity of the *Chryseobacterium* species was standardised using Z-scores which were calculated as: Z = colony diameter (mm)/(colony diameter (mm) + zone size (mm)). Analysis of variance (ANOVA) was performed on all the data using the Tukey–Kramer multiple comparison test at α = 0.05 [49] to determine any significant differences in treatment means.

## 3. Results

### 3.1. Qualitative and Quantitative Enzyme Production

The qualitative (absence/presence) and quantitative (Z-score) analysis of enzyme production are indicated in Table 2. The determination of hyaluronidase and chondroitinase activity were performed only qualitatively, as the zones of clearance were washed off by the acetic acid which formed a non-degradable substrate that precipitated as a conjugate with the albumin making it difficult to measure the zones. Lecithinase activity was also only qualitatively determined as the colonies spread over the plates.

In this study, all 38 strains tested showed capability to break down the haemoglobin of red blood cells, which was the first most important indicator of its potentially pathogenic characteristics [11]. Seventy-six percent (29/38) of the species in this study showed α-haemolysis, while the rest showed β-haemolysis (Table 2). Of the human clinically isolated and diseased fish isolates, *C. balustinum* (fish), *C. gleum* (human), *C. indologenes* (human) and *C. scophthalmum* (fish) showed α-haemolysis, while *E. meningoseptica* (human) showed β-haemolysis. Alpha-haemolysis, was indicative of a partial break down of red blood cells, and it left a greenish colour caused by the presence of biliverdin, a by-product of the breakdown of haemoglobin [10]. Beta-haemolysis was indicative of complete breakdown of the haemoglobin and red blood cells, leaving a clear zone around the bacterial growth. None of the species tested showed γ-haemolysis, which is indicative of no haemolysis [10].

The ability of an organism to grow at 37 °C may be indicative that the organism can survive and grow in the human body at a temperature of 37 °C and may, therefore, cause disease. In this study, 23/38 *Chryseobacterium* strains were able to grow at 37 °C. However, the absence of this characteristic (Table 2) in *C. balustinum*, *C. carnipullorum*, *C. formosense*, *C. gregarium*, *C. hispanicum*, *C. indoltheticum*, *C. jeonii*, *C. joostei*, *C. luteum*, *C. piscium*, *C. scophthalmum*, *C. shigense*, *C. soli*, *C. ureilyticum* and *C. vrystaatense*, is not indicative that they are not pathogenic. They might not be pathogenic to humans, but they may still cause disease or spoilage in animals, fish and plants. This was the case with *C. balustinum* [22] and *C. scophthalmum* [28], which were isolated from diseased fish.

Lecithinase was produced by 55% (21/38) of the strains evaluated in this study. Lecithinase (phospholipolytic) production is used as an indicator of food toxicity. The phospholipid lecithin is one of the main components of cell membranes and can be degraded by lecithinase to produce a diglyceride and phosphorylcholine, causing toxicity. Lecithinase can damage reproductive tract tissues and cause haemolysis and membrane disruption, leading to cell lysis [50]. The majority of the environmental and human pathogenic strains (*C. gleum*, *C. indologenes* and *E. meningoseptica*) in this study showed this characteristic, indicating their ability to cause pathogenic symptoms in humans and plants. However, the fish pathogens, *C. balustinum*, *C. hominis* and *C. scophthalmum;* the food isolates, *C. bovis*, *C. joostei*, *C. shigense* and C. *vrystaatense*; and the water isolates, *C. aquaticum* and *C. koreense* (Table 2) will not be able to produce lecithinase toxicity in food and water sources.

Hyaluronidase was produced by all the strains evaluated in this study except for *C. balustinum* and *C. piscium*. Hyaluronidase degrades hyaluronic acid, which is present in tissues throughout the body, including the bones and joints. The ability of bacteria to degrade hyaluronic acid is regarded as a virulence factor, enabling penetration of hyaluronidase-producing organisms into tissues rich in hyaluronic acid, creating an advantage for establishing growth of these organisms into the body [51]. All the human opportunistic pathogens, environmental, water and food isolates in this study could therefore cause harm to human tissue. Only two fish isolates, *C. balustinum* and *C. piscium*, did not show this characteristic.

Chondroitinase was produced by 87% (33/38) of the strains evaluated with only *C. balustinum*, *C. formosense*, *C. hispanicum*, *C. jeonii* and *C. piscium* not having this characteristic (Table 2). Bacterial chondroitinase may be associated with bacterial pathogenicity by catalysing the hydrolysis of chondroitin sulphate, a constituent of the extracellular matrix of cartilage, and may increase tissue permeability to invade the cartilage tissue [52]. It may also degrade animal residue in nature and may serve in addition as a tool in medical and biochemical studies on connective tissues [53]. The majority of the human and fish pathogens and environmental, food and water isolates in this study showed this characteristic.

None of the strains evaluated in this study produced fibrinolysin, which indicated that they do not have the capability to act as fibrinolytic or thrombolytic agents that convert plasminogen to plasmin and lyse blood clots by breaking down the fibrin contained in the clot [54].

In order for a microorganism to be considered pathogenic or virulent, it should produce at least two or more extracellular enzymes [11]. All the species tested in this study produced at least two enzymes, except for *C. balustinum* and *C. piscium*. Both these species were isolated from diseased fish (Table 1), which might indicate that these two species use other mechanisms to cause disease in fish and will not be pathogenic to humans. Therefore, there was an increased possibility for *C. aquafrigidense*, *C. carnipullorum*, *C. daecheongense*, *C. daeguense*, *C. defluvii*, *C. flavum*, *C. gleum*, *C. gregarium*, *C. indologenes*, “*C. massiliense*”, *C. oranimense*, *C. taenense*, *C. taichungense*, *C. taiwanense* and *C. wanjuense* to be pathogenic by possessing 89% (8/9) of enzymes (Table 2) from groups that supply nutrients for their survival in the host (e.g., proteases and lipases) and those that aid the organisms to enter the host and pass through its tissue (DNase and elastase) [10,11]. *Chryseobacterium gleum* and *C. indologenes*, which have been reported by many authors [55,56] to be common strains of clinical interest, were found to contain all the virulence factors evaluated in this study, except for fibrinolysin production, while *E. meningoseptica*, which causes meningitis in humans [47] produced 7/9 enzymes evaluated in this study. One of the fish pathogens in this study, *C. scophthalmum*, only produced gelatinase and DNase, enzymes that aid in entering of the host and passing through the tissues [10,11].

*C. carnipullorum* which was isolated from a food source [24], *C. gambrini* which was isolated from the steel surface of a beer bottling plant [35] and *C. soldanellicola* which was isolated from the roots of sand dune plants [40] produced 8/9, 7/9 and 7/9, respectively, of the enzymes tested. This might be indicative that these species might be pathogenic to humans, although *C. carnipullorum* did not have the ability to grow at 37 °C. The presence of the amount of different enzymes might also indicate that these species play a role in the spoilage of food or the breaking down of complex carbohydrates in sand for root growth stimulation.

The quantitative enzyme production results indicated that the opportunistic human pathogens, *C. gleum*, *C. indologenes* and *E. meningoseptica*, produced proteases as a pathogenic characteristic, while the fish pathogens in this study rather produced elastase (*C. balustinum* and *C. scophthalmum*), gelatinase (*C. piscium* and *C. scophthalmum*) and DNase (*C. scophthalmum*). *Chryseobacterium soldanellicola*, the environmentally isolated strain in this study, produced all of the enzymes with the best production for protease, gelatinase and elastase.

*Chryseobacterium gleum* had a significantly (*p* < 0.001) higher protease production (0.378) than *C. indologenes* (0.632) and *Elizabethkingia meningoseptica* (0.916). *Chryseobacterium soldanellicola* had the second highest protease production, which was significantly (*p* < 0.001) higher than the pathogens *C. indologenes* and *E. meningoseptica*. Proteases hydrolyse the peptide bond present in the polypeptide chain of amino acids [57]. The species evaluated in this study, however, did not use lipase to produce disease in humans or fish and do not play a major role in the environment or food spoilage since their Z-score values were lower than 0.500 (Table 2).

In this study, *C. molle* was able to produce gelatinase, which is in accordance with the fact that it was isolated from the biofilm of a conveyer belt in a beer bottling plant [35]. Bacterial growth as a biofilm on solid surfaces is strongly associated with the development of human infections. Some bacteria, e.g., *Enterococcus faecalis*, control biofilm development through the production of gelatinase [58]. All of the organisms, with high levels of gelatinase production, as shown in Table 2, were isolated from either food or environmental sources. The ability of these organisms to produce gelatinases in these sources may be an indication of their survival strategies and may possibly play a role in the spoilage potential in food sources.

*Chryseobacterium scophthalmum*, a fish pathogen, and *C. gleum*, a human pathogen, had the significantly (*p* < 0.001) highest DNase production activities compared to *C. oranimense* (0.393), C. *gregarium* (0.421) and *C. koreense* (0.475) (Table 2). DNases play an important role in DNA utilization, nutrient cycling, the attachment and stability of the biofilm matrix and are well known for being able to break up biofilms [59]. The DNase production activities exhibited by *C. scophthalmum* and *C. gleum* could be indicative that this enzyme is used to cause disease.

Elastase was produced by the biggest number of species evaluated in this study (11/38), but none of them produced Z-scores lower than 0.400 (Table 2). *Chryseobacterium daecheongense*, *C. daeguense*, *C. defluvii*, *C. gleum*, *C. indologenes*, *C. luteum*, “*C. massiliense*”, *C. oranimense*, *C. soldanellicola*, *C. taenense* and *C. wanjuense* produced elastases (Z-scores of < 0.500) capable of solubilizing fibrous elastin and may play a pathologic role in pulmonary emphysema, cystic fibrosis, infections, inflammation and atherosclerosis [60].

The *Chryseobacterium* species that produced more than two enzymes with high enzyme production (Z-scores < 0.5) were *C. gleum* and *C. soldanellicola* (Table 2). Since *C. soldanellicola* was isolated from the roots of sand dune plants, it is speculated that the high production capabilities of this organism help with the survival of the sand dune plants in breaking down material in order for the plants to survive. It might also indicate that this species might be pathogenic in humans.

### 3.2. Antimicrobial Resistance/Susceptibility

*Chryseobacterium aquafrigidense*, *C. bovis*, *C. gambrini* and *C. taichungense* were susceptible to all ten antimicrobials evaluated, as they had zone sizes of 50 mm (Table 3). Most *Chryseobacterium* species are known to be resistant to a wide range of antimicrobials [9]. In this study, *C. gleum*, *C. indologenes*, *C. joostei*, *C. daecheongense*, *C. daeguense*, *C. shigense*, *C. soldanellicola*, *C. soli*, *C. ureilyticum*, *C. vrystaatense*, *C. wanjuense* and *E. meningoseptica* were resistant to most of the antimicrobials evaluated. The findings in this study were also in agreement with those of other authors [61,62]. In this study, *C. indologenes* (isolated from soil, water and the clinical environment) and *C. joostei* (isolated from raw milk) were resistant to most (8/10) of the antimicrobials. Resistance to antimicrobials will make treatment of infections caused by these organisms difficult.

Of the *Chryseobacterium* species, 58% (22/38) were resistant to cephalothin, neomycin and chloramphenicol, 53% (20/38) were resistant to amoxicillin while 50% (19/38) were resistant to ampicillin (Table 4). Conversely, 87% (33/38) of the species were susceptible to trimethoprim, 82% (31/38) were susceptible to oxytetracycline, 76% (29/38) were susceptible to vancomycin, 74% (28/38) were susceptible to tetracycline and 55% (21/38) were susceptible to streptomycin.

Therefore, the class of antimicrobials that was more effective at suppressing the survival of *Chryseobacterium* strains were the folic acid synthesis inhibitors (trimethoprim) with the protein synthesis inhibitors (oxytetracycline and tetracycline) and the cell wall synthesis inhibitors class (vancomycin) in second and third places, respectively. This finding was consistent with the findings of other authors [63] who reported trimethoprim-sulfamethoxazole as one of the most active agents against *C. indologenes*, which is one of the currently known opportunistic pathogens.

### 3.3. Disinfectant Resistance/Susceptibility

The results of the resistance of the 14 *Chryseobacterium* species against four commercially available disinfectants are given in Table 5. The results are expressed as the minimum inhibitory concentration (MIC) of each disinfectant, meaning that it is the lowest concentration of a disinfectant where the test organism was inhibited (no growth). All positive controls showed growth (no inhibition), and the negative controls did not show any growth (inhibition).

The most effective of the four disinfectants against the *Chryseobacterium* species evaluated was Disinfectant 4, with poly-dimethyl ammonium chloride as the active ingredient. All 14 species in this study were susceptible to Detergent 4, with MICs of < 0.06% (Table 5). The second most effective disinfectant was Disinfectant 2 with benzalkonium chloride as the active ingredient. *Chryseobacterium balustinum*, *C. formosense*, *C. indoltheticum*, *C. piscium*, *C. scophthalmum*, *C. soldanellicola*, *C. taenense* and *C. taichungense* were susceptible to Detergent 2, whereas *C. joostei* was the most resistant to Detergent 2, with a MIC of 0.50%. *Chryseobacterium daecheongense*, *C. gleum*, *C. indologenes*, *C. shigense* and *C. vrystaatense* were moderately resistant, with MICs between 0.25% and 0.13%.

Disinfectant 1 with chloroxylenol as an active ingredient was the third most effective of the tested disinfectants. All 14 strains were moderately susceptible (MICs between 0.13% and 0.25%) to Detergent 1, while *C. gleum*, *C. indologenes*, *C. joostei*, *C. piscium*, *C. scophthalmum* and *C. vrystaatense* were resistant (MICs between 0.50% and 1.00%) (Table 5). Disinfectant 3 (chlorhexidine gluconate and cetrimide as active ingredients) was deemed the least effective of the tested disinfectants.

From these results, it seems as if the *Chryseobacterium* species were more resistant to the disinfectants used on wounds (Disinfectants 1 and 3) than to the surface disinfectants (Disinfectants 2 and 4). This could be problematic, especially in the case of *C. indologenes* and *C. gleum*, which have been previously associated with wound infections [55,56].

## 4. Conclusions

This study is the first to indicate the potential pathogenic characteristics and control by disinfectants of *Chryseobacterium* species from environmental, food, fish, water and clinical sources. Most of the species evaluated in this study showed a variety of virulence characteristics. All 38 species evaluated were either able to break down haemoglobin partly or completely. Five out of the six water isolates, only *C. oranimense* (isolated from raw cow milk) of the nine food isolates, 11/18 environmental isolates and all the five clinical isolates were able to grow at 37 ^o^C, which was an indication that they will be able to grow at the human body temperature of 37 ^o^C. However, this characteristic cannot be used as a single indicator of virulence of an isolate.

When compared to the clinically isolated *C. gleum* and *C. indologenes*, the virulence enzyme production indicated that isolates from food sources (*C. carnipullorum*) and the environment (*C. gambrini* and *C. soldanellicola*) produced the highest number of enzymes, 8/9 and 7/9 enzymes, respectively. In this regard, these isolates could be potential human pathogens. The fish isolates, e.g., *C. piscium*, will not be pathogenic to humans, but when compared to the fish pathogens, *C. balustinum* and *C. scophthalmum* might be pathogenic to fish.

Of the 38 isolates, *C. indologenes* and *C. joostei* were resistant to most (8/10) of the antimicrobials evaluated. The isolates that were regarded as pathogenic by the amount of enzyme production, *C. carnipullorum*, *C. gambrini* and *C. soldanellicola*, were resistant to 6/10, 0/10 and 4/10 of the antibiotics tested, respectively. This might indicate that the food-isolated *C. joostei* and *C. carnipullorum*, when acting as opportunistic pathogens, might prove difficult to treat. The food isolates, *C. joostei* and *C. carnipullorum*, could be susceptible to trimethoprim and vancomycin, respectively. The antimicrobials that could be used as treatment for pathogenesis caused by most of the *Chryseobacterium* species in this study were oxytetracycline and trimethoprim, with >80% of the isolates being susceptible to these antimicrobials.

For the control of *Chryseobacterium* growth by disinfectants, those containing the active ingredient poly-dimethyl ammonium chloride could serve as the best option for decontamination, followed by those containing the active ingredient benzalkonium chloride.

The implications of this study are that a combination of virulence factors should be used in the evaluation of the pathogenicity of isolates and that all *Chryseobacterium* isolates from food, especially, should be tested for pathogenicity in the future. The antimicrobials and disinfectants recommended in this study will be able to control the growth of these organisms.

## Figures and Tables

**Table 1:** Type strains used in the determination of pathogenic characteristics of *Chryseobacterium* species from food and environmental sources. Strains indicated in bold are the reference pathogenic strains used in this study. * Strains selected for the disinfectant resistance studies.

*Chryseobacterium* Strains Used	Culture Collection Number	Source of Isolation	Reference
**Water**			
*C. aquafrigidense*	KCTC 12484 ^T^	Cooled water from an oxygen-producing plant	[16]
*C. aquaticum*	KCTC 12483 ^T^	Water reservoir	[17]
* *C. daecheongense*	DSM 15235 ^T^	Freshwater lake sediment	[18]
*C. daeguense*	KCTC 12841 ^T^	Wastewater of a textile dye works	[19]
*C. hispanicum*	KCTC 22104 ^T^	Drinking water distribution system	[20]
*C. koreense*	KCTC 12107 ^T^	Natural mineral water	[21]
**Food**			
* *C. balustinum*	NCTC 11212 ^T^	Diseased freshwater fish	[22]
*C. bovis*	LMG 24227 ^T^	Raw cow milk	[23]
*C. carnipullorum*	LMG 26732 ^T^	Raw chicken meat	[24]
* *C. joostei*	LMG 18212 ^T^	Raw milk	[25]
*C. oranimense*	DSM 19055 ^T^	Raw cow milk	[26]
* *C. piscium*	CCUG 51923 ^T^	Marine fish	[27]
* *C. scophthalmum*	LMG 13028 ^T^	Diseased turbot fish gills	[28]
* *C. shigense*	DSM 17126 ^T^	Lactic acid beverage	[29]
* *C. vrystaatense*	LMG 22846 ^T^	Raw chicken meat	[30]
**Environmental**			
*C. caeni*	DSM 17710 ^T^	Bioreactor sludge	[31]
*C. defluvii*	DSM 14219 ^T^	Activated sludge	[32]
*C. flavum*	KCTC 12483 ^T^	Herbicide polluted soil	[33]
* *C. formosense*	CCUG 49271 ^T^	Rhizosphere of garden lettuce	[34]
*C. gambrini*	DSM 18014 ^T^	Steel surface of a beer bottling plant	[35]
*C. gregarium*	LMG 24052 ^T^	Decaying plant material	[36]
*C. hungaricum*	DSM 19684 ^T^	Kerosene contaminated soil	[37]
* *C. indoltheticum*	ATCC 27950 ^T^	Marine mud	[2]
*C. jeonii*	KCTC 12226 ^T^	Moss near penguin habitat	[38]
*C. luteum*	LMG 23785 ^T^	Phyllosphere of grasses	[39]
*C. molle*	DSM 18016 ^T^	Biofilm of a conveyor of a beer-bottling plants	[35]
* *C. soldanellicola*	CCUG 52904 ^T^	Roots of sand-dune plants (*Calystegia soldanella*)	[40]
*C. soli*	DSM 19298 ^T^	Soil	[41]
* *C. taeanense*	CCUG 52900 ^T^	Roots of sand-dune plants (*Elymus mollis*)	[40]
* *C. taichungense *	CCUG 50001 ^T^	Soil	[42]
*C. taiwanense*	LMG 23355 ^T^	Farmland soil	[43]
*C. ureilyticum*	CCUG 18017 ^T^	Steel surface of a beer-bottling plant	[35]
*C. wanjuense*	KCTC 22055 ^T^	Greenhouse soil cultured with lettuce	[44]
**Clinical**			
* *C. gleum* (Type species)	NCTC 11432 ^T^	Human vaginal swab	[2]
*C. hominis*	DSM 19326 ^T^	Clinical blood isolates/Kidneys of a pufferfish	[45]
“Candidatus *C. massiliense*”	CCUG 51329 ^T^	Human nasal swab	[46]
* *C. indologenes*	LMG 8337 ^T^	Soil/water/clinical origin	[2]
***Elizabethkingia meningoseptica***	NCTC 10116 ^T^	Cerebrospinal fluid of premature infant	[47]

ATCC, American Type Culture Collection (USA); LMG, Laboratorium voor Microbiologie (Ghent, Belgium); CCUG, Culture Collection, University of Gothenburg (Sweden); DSM, Deutsche Sammlung von Mikroorganismen und Zellkulturen (Germany); KCTC, Korean Collection for Type Cultures (Korea); NCTC, National Collection of Type Cultures (England); ^T^, type strain.

**Table 2:** Qualitative (absence/presence) analysis of haemolysis, growth at 37 °C, production of lecithinase, hyaluronidase, chondroitinase and fibrinolysin, and quantitative analysis (Z-score values) production of protease, lipase, gelatinase, DNase and elastase of the 37 *Chryseobacterium* species and *Elizabethkingia meningoseptica* evaluated in this study. α, alpha haemolysis; β, beta haemolysis; +, present/positive; −, absent/negative; (+), weakly positive; N/A. not applicable. Species in bold are the control strains, isolated from human clinical samples or diseased fish. Highest enzyme production (Z-score < 0.5) is indicated in bold. *n* = 6.

Type Species	Haemolysis	Growth at 37 °C	Lecithinase	Hyaluronidase	Chondroitinase	Fibrinolysin	Protease	Lipase	Gelatinase	DNase	Elastase
*C. aquafrigidense*	β	+	+	+	+	−	0.939 ^g^	0.914 ^cdefgh^	0.942 ^hij^	0.855 ^fghij^	0.809 ^g^
*C. aquaticum*	α	+	−	+	+	−	0.909 ^fg^	0.937 ^ghij^	0.882 ^h^	0.886 ^hijk^	0.589 ^ef^
***C. balustinum***	α	−	−	−	−	−	1.000 ^g^	1.000 ^k^	1.000 ^j^	1.000 ^k^	0.577 ^ef^
*C. bovis*	α	+	−	+	+	−	0.585 ^c^	0.903 ^bcdefg^	0.902 ^hi^	0.949 ^jk^	1.000 ^h^
*C. caeni*	β	+	(+)	+	+	−	1.000 ^g^	0.948 ^hij^	0.922 ^hi^	0.901 ^ijk^	1.000 ^h^
*C. carnipullorum*	α	−	(+)	+	(+)	−	1.000 ^g^	0.953 ^ij^	0.937 ^hi^	0.833 ^fghij^	0.584 ^ef^
*C. daecheongense*	α	+	+	+	+	−	0.511 ^bc^	0.888 ^abcd^	0.912 ^hi^	0.868 ^ghijk^	**0.419** ^ab^
*C. daeguense*	α	+	+	+	+	−	0.547 ^bc^	0.870 ^ab^	0.879 ^h^	0.713 ^def^	**0.444** ^abc^
*C. defluvii*	α	+	+	+	+	−	0.751 ^de^	0.860 ^a^	0.937 ^hij^	0.505 ^bc^	**0.448** ^abc^
*C. flavum*	α	+	+	+	+	−	0.504 ^abc^	0.924 ^efghij^	0.759 ^fg^	0.819 ^efghij^	0.506 ^abcde^
*C. formosense*	β	−	−	(+)	−	−	1.000 ^g^	0.957 ^j^	**0.439** ^d^	1.000 ^k^	1.000 ^h^
*C. gambrini*	α	+	(+)	+	(+)	−	0.529 ^bc^	0.936 ^ghij^	0.745 ^f^	0.729 ^defg^	1.000 ^h^
***C. gleum***	α	+	+	+	+	−	**0.378** ^a^	0.930 ^fghij^	0.958 ^ij^	**0.268** ^a^	**0.414** ^a^
*C. gregarium*	α	−	+	+	+	−	0.575 ^bc^	0.892 ^abcde^	0.911 ^hi^	**0.421** ^b^	0.513 ^cde^
*C. hispanicum*	α	−	(+)	(+)	−	−	1.000 ^g^	1.000 ^k^	1.000 ^j^	1.000 ^k^	1.000 ^h^
*C. hominis*	β	+	−	+	+	−	1.000 ^g^	0.951 ^ij^	0.934 ^hi^	0.916 ^ijk^	1.000 ^h^
*C. hungaricum*	β	+	−	+	(+)	−	1.000 ^g^	0.947 ^hij^	0.931 ^hi^	1.000 ^k^	1.000 ^h^
***C. indologenes***	α	+	(+)	+	(+)	−	0.632 ^cd^	0.893 ^abcde^	0.917 ^hi^	0.687 ^de^	**0.446** ^abc^
*C. indotheticum*	α	−	−	+	+	−	0.507 ^abc^	1.000 ^k^	0.957 ^ij^	0.746 ^defgh^	0.659 ^f^
*C. jeonii*	α	−	(+)	(+)	−	−	1.000 ^g^	1.000 ^k^	1.000 ^j^	1.000 ^k^	1.000 ^h^
*C. joostei*	α	−	−	(+)	(+)	−	1.000 ^g^	1.000 ^k^	**0.432** ^cd^	1.000 ^k^	0.508 ^bcde^
*C. koreense*	β	+	−	+	+	−	1.000 ^g^	0.882 ^abc^	0.813 ^g^	**0.475** ^b^	1.000 ^h^
*C. luteum*	α	−	−	(+)	(+)	−	1.000 ^g^	1.000 ^k^	**0.375** ^abc^	1.000 ^k^	**0.456** ^abc^
“*C. massiliense*”	β	+	+	+	+	−	0.555 ^bc^	0.895 ^abcdef^	0.907 ^hi^	0.779 ^efghi^	**0.441** ^abc^
*C. molle*	β	+	(+)	+	+	−	1.000 ^g^	1.000 ^k^	**0.426** ^bcd^	1.000 ^k^	1.000 ^h^
*C. oranimense*	α	+	+	+	+	−	0.941 ^g^	0.960 ^j^	0.914 ^hi^	**0.393** ^ab^	**0.475** ^abcd^
*C. piscium*	α	−	−	−	−	−	1.000 ^g^	1.000 ^k^	0.581 ^e^	1.000 ^k^	1.000 ^h^
***C. scophthalmum***	α	−	−	(+)	+	−	1.000 ^g^	1.000 ^k^	**0.365** ^ab^	**0.256** ^a^	0.825 ^g^
*C. shigense*	α	−	−	(+)	+	−	1.000 ^g^	1.000 ^k^	1.000 ^j^	1.000 ^k^	0.555 ^de^
*C. soldanellicola*	α	+	−	+	+	−	**0.449** ^ab^	0.918 ^cdefghi^	**0.333** ^a^	0.684 ^de^	**0.423** ^abc^
*C. soli*	α	−	−	(+)	(+)	−	1.000 ^g^	1.000 ^k^	1.000 ^j^	1.000 ^k^	0.578 ^ef^
*C. taeanense*	α	+	+	+	+	−	0.807 ^ef^	0.887 ^abcd^	0.885 ^h^	0.843 ^fghij^	**0.444** ^abc^
*C. taichungense*	α	+	(+)	+	+	−	0.625 ^cd^	0.892 ^abcde^	0.919 ^hi^	0.820 ^efghij^	0.502 ^abcde^
*C. taiwanense*	α	+	+	+	+	−	0.589 ^c^	0.897 ^bcdef^	0.920 ^hi^	0.877 ^hijk^	0.560 ^d^e
*C. ureilyticum*	α	−	−	+	+	−	1.000 ^g^	1.000 ^k^	1.000 ^j^	0.773 ^defghi^	0.514 ^cde^
*C. vrystaatense*	α	−	−	(+)	+	−	1.000 ^g^	1.000 ^k^	**0.478** ^d^	1.000 ^k^	1.000 ^h^
*C. wanjuense*	α	+	+	+	+	−	0.613 ^c^	0.868 ^ab^	0.913 ^hi^	0.894 ^ijk^	**0.478** ^abcd^
***E. meningoseptica***	β	+	+	+	+	−	0.916 ^fg^	0.919 ^defghi^	0.910 ^hi^	0.635 ^cd^	1.000 ^h^
**Significance level**	N/A	N/A	N/A	N/A	N/A	N/A	*p* < **0.001**	*p* < **0.001**	*p* < **0.001**	*p* < **0.001**	*p* < **0.001**

Means with different superscripts in the same column differed significantly (*n* = 6). N/A, not analysed.

**Table 3:** Resistance/susceptibility patterns for the 37 *Chryseobacterium* species and *Elizabethkingia meningoseptica* used in this study. Values are the zone sizes given in millimetres. Means with different superscripts in the same column differed significantly (*n* = 6).

Organism	Ampicillin	Amoxicillin	Vancomycin	Cephalothin	Neomycin	Tetracycline	Oxytetracycline	Streptomycin	Chloramphenicol	Trimethoprim
*C. aquafrigidense*	50.000 ^l^	50.000 ^j^	50.000 ^p^	50.000 ^j^	50.000 ^m^	50.000 ^q^	50.000 ^p^	50.000 ^r^	50.000 ^j^	50.000 ^o^
*C. aquaticum*	14.320 ^c^	15.127 ^de^	17.233 ^cdefg^	13.713 ^d^	10.267 ^efgh^	28.377 ^j^	24.337 ^kl^	13.117 ^defg^	25.123 ^h^	41.127 ^mn^
*C. hominis*	40.880 ^jk^	41.650 ^i^	23.903 ^kl^	49.030 ^j^	15.977 ^i^	46.640 ^p^	6.170 ^a^	26.520 ^no^	6.170 ^a^	6.170 ^a^
*C. balustinum*	24.663 ^de^	24.177 ^f^	23.800 ^kl^	28.767 ^h^	14.623 ^i^	32.427 ^kl^	30.630 ^m^	19.830 ^m^	23.287 ^g^	38.087 ^lmn^
*C. bovis*	50.000 ^l^	50.000 ^j^	50.000 ^p^	50.000 ^j^	50.000 ^m^	50.000 ^q^	50.000 ^p^	50.000 ^r^	50.000 ^j^	50.000 ^o^
*C. caeni*	50.000 ^l^	50.000 ^j^	50.000 ^p^	50.000 ^j^	50.000 ^m^	50.000 ^q^	50.000 ^p^	27.620 ^p^	50.000 ^j^	50.000 ^o^
*C. carnipullorum*	6.170 ^a^	6.170 ^a^	21.067 ^ijk^	6.170 ^a^	6.170 ^a^	6.170 ^a^	15.397 ^cde^	13.410 ^efgh^	6.170 ^a^	12.230 ^ab^
*C. daecheongense*	6.170 ^a^	6.170 ^a^	17.910 ^efgh^	6.170 ^a^	6.170 ^a^	6.170 ^a^	21.010 ^hijk^	12.553 ^def^	17.140 ^f^	36.843 ^jklmn^
*C. daeguense*	6.170 ^a^	6.170 ^a^	17.933 ^efgh^	6.170 ^a^	6.170 ^a^	6.170 ^a^	22.010 ^ijkl^	16.743 ^ijk^	10.373 ^cd^	39.983 ^mn^
*C. defluvii*	50.000 ^l^	50.000 ^j^	29.380 ^m^	6.170 ^a^	40.143 ^m^	6.170 ^a^	6.170 ^a^	39.387 ^q^	6.170 ^a^	35.993 ^ijklmn^
*C. flavum*	6.170 ^a^	6.170 ^a^	12.290 ^a^	6.170 ^a^	10.990 ^gh^	11.480 ^d^	12.733 ^bcd^	6.170 ^a^	6.170 ^a^	27.770 ^defgh^
*C. formosense*	11.200 ^bc^	11.383 ^bcd^	21.607 ^jk^	11.097 ^c^	6.170 ^a^	24.580 ^i^	22.533 ^jkl^	11.237 ^bcd^	9.300 ^bc^	36.330 ^ijklmn^
*C. gambrini*	50.000 ^l^	50.000 ^j^	50.000 ^p^	50.000 ^j^	50.000 ^m^	50.000 ^q^	50.000 ^p^	50.000 ^r^	50.000 ^j^	50.000 ^o^
*C. gleum*	6.170 ^a^	6.170 ^a^	18.050 ^efgh^	6.170 ^a^	9.833 ^defgh^	15.627 ^e^	15.780 ^cdef^	17.107 ^jk^	8.277 ^b^	32.163 ^ghijkl^
*C. gregarium*	11.627 ^bc^	10.170 ^abc^	18.933 ^fghij^	10.993 ^c^	9.327 ^cdefg^	28.430 ^j^	25.357 ^l^	15.197 ^hij^	8.257 ^b^	30.870 ^fghijk^
*C. hispanicum*	31.670 ^fg^	33.950 ^g^	16.030 ^cde^	25.450 ^g^	19.000 ^j^	38.600 ^n^	33.340 ^mn^	30.483 ^p^	30.193 ^i^	39.037 ^mn^
*C. hungaricum*	37.193 ^ij^	39.747 ^hi^	33.150 ^n^	45.270 ^i^	8.397 ^bcd^	43.527 ^o^	40.567 ^o^	13.987 ^fgh^	22.477 ^g^	37.340 ^klmn^
*C. indologenes*	6.170 ^a^	6.170 ^a^	18.037 ^efgh^	6.170 ^a^	6.170 ^a^	8.047 ^ab^	9.483 ^ab^	6.170 ^a^	9.533 ^bcd^	16.623 ^bc^
*C. indotheticum*	8.787 ^ab^	7.607 ^ab^	20.183 ^hij^	7.778 ^ab^	7.378 ^ab^	21.887 ^gh^	19.067 ^fghi^	9.333 ^b^	10.830 ^d^	25.117 ^def^
*C. jeonii*	32.870 ^gh^	35.247 ^g^	14.790 ^abcd^	26.997 ^gh^	18.060 ^j^	35.160 ^m^	32.063 ^mn^	25.133 ^n^	26.427 ^h^	38.187 ^lmn^
*C. joostei*	6.170 ^a^	6.170 ^a^	16.207 ^cdef^	6.170 ^a^	6.170 ^a^	9.237 ^bc^	10.527 ^b^	9.833 ^bc^	6.170 ^a^	29.673 ^efghi^
*C. koreense*	41.660 ^k^	46.433 ^j^	34.810 ^no^	46.130 ^i^	33.533 ^k^	47.700 ^p^	18.410 ^efgh^	50.000 ^r^	50.000 ^j^	10.907 ^ab^
*C. luteum*	12.953 ^c^	12.283 ^cd^	13.013 ^ab^	9.987 ^bc^	10.057 ^defgh^	27.283 ^j^	22.953 ^jkl^	9.783 ^bc^	6.170 ^a^	34.297 ^hijklm^
“*C. massiliense*”	28.217 ^ef^	50.000 ^j^	36.747 ^o^	27.307 ^gh^	50.000 ^m^	43.253 ^o^	50.000 ^p^	50.000 ^r^	50.000 ^j^	50.000 ^o^
*C. molle*	36.170 ^hi^	37.500 ^gh^	20.263 ^hij^	18.203 ^e^	17.810 ^j^	34.150 ^lm^	34.493 ^n^	19.917 ^m^	25.820 ^h^	42.747 ^n^
*C. oranimense*	6.170 ^a^	6.170 ^a^	12.890 ^abc^	6.170 ^a^	7.770 ^bc^	14.430 ^e^	16.350 ^cde^	11.910 ^def^	6.170 ^a^	23.410 ^de^
*C. piscium*	13.843 ^c^	13.003 ^cd^	17.453 ^cdefg^	10.210 ^c^	8.730 ^bcde^	22.293 ^h^	20.543 ^ghij^	11.717 ^cde^	8.540 ^b^	22.167 ^cd^
*C. scophthalmum*	11.877 ^bc^	9.467 ^abc^	19.580 ^ghij^	10.030 ^bc^	7.990 ^bc^	22.727 ^hi^	21.187 ^hijk^	10.383 ^bc^	9.013 ^bc^	27.663 ^defgh^
*C. shigense*	6.170 ^a^	6.170 ^a^	21.247 ^ijk^	6.170 ^a^	8.610 ^bcde^	19.343 ^f^	16.420 ^ef^	14.473 ^fgh^	6.170 ^a^	24.947 ^def^
*C. soldanellicola*	8.237 ^ab^	6.170 ^a^	15.803 ^bcde^	9.913 ^bc^	11.363 ^h^	24.490 ^i^	23.397 ^jkl^	11.620 ^cde^	8.543 ^b^	30.313 ^fghij^
*C. soli*	6.170 ^a^	6.170 ^a^	24.897 ^l^	6.170 ^a^	14.787 ^i^	19.903 ^fg^	16.167 ^def^	17.747 ^kl^	6.170 ^a^	26.240 ^defg^
*C. taeanense*	21.187 ^d^	17.533 ^e^	18.470 ^efghi^	22.273 ^f^	8.963 ^bcdef^	30.830 ^k^	30.300 ^m^	19.697 ^lm^	6.170 ^a^	15.527 ^bc^
*C. taichungense*	50.000 ^l^	50.000 ^j^	50.000 ^p^	50.000 ^j^	50.000 ^m^	50.000 ^q^	50.000 ^p^	50.000 ^r^	50.000 ^j^	50.000 ^o^
*C. taiwanense*	50.000 ^l^	50.000 ^j^	26.007 ^l^	50.000 ^j^	50.000 ^m^	50.000 ^q^	50.000 ^p^	50.000 ^r^	50.000 ^j^	50.000 ^o^
*C. ureilyticum*	6.170 ^a^	6.170 ^a^	16.907 ^cdefg^	6.170 ^a^	9.917 ^defgh^	10.427 ^cd^	10.647 ^b^	12.573 ^def^	6.170 ^a^	14.563 ^b^
*C. vrystaatense*	6.170 ^a^	6.170 ^a^	18.177 ^efgh^	6.170 ^a^	10.570 ^fgh^	17.143 ^e^	17.540 ^efg^	11.323 ^bcd^	6.170 ^a^	14.590 ^b^
*C. wanjuense*	6.170 ^a^	6.170 ^a^	17.540 ^defgh^	7.437 ^a^	6.170 ^a^	23.397 ^hi^	25.110 ^l^	15.040 ^ghi^	8.500 ^b^	15.593 ^bc^
*E. meningoseptica*	50.000 ^l^	50.000 ^j^	24.867 ^l^	50.000 ^j^	50.000 ^m^	9.527 ^bcd^	12.610 ^bc^	50.000 ^r^	13.347 ^e^	50.000 ^o^
Significance *p* <	0.001	0.001	0.001	0.001	0.001	0.001	0.001	0.001	0.001	0.001

**Table 4:** Percentage resistance and susceptibility of *Chryseobacterium* species and *Elizabethkingia meningoseptica* to antimicrobials.

Antimicrobial	Resistant (%)	Intermediate (%)	Susceptible (%)
Ampicillin (10 μg)	50.00 (19/38)	5.26 (2/38)	44.74 (17/38)
Amoxicillin (10 μg)	52.63 (20/38)	2.60 (1/38)	44.77 (17/38)
Vancomycin (30 μg)	7.89 (3/38)	15.79 (6/38)	76.32 (29/38)
Cephalothin (30 μg)	57.89 (22/38)	0.00 (0/38)	42.11 (16/38)
Neomycin (30 μg)	57.89 (22/38)	0.00 (0/38)	42.11 (16/38)
Tetracycline (30 μg)	21.00 (8/38)	5.26 (2/38)	73.74 (28/38)
Oxytetracycline (30 μg)	13.16 (5/38)	5.26 (2/38)	81.58 (31/38)
Streptomycin (25 μg)	21.00 (8/38)	23.68 (9/38)	55.32 (21/38)
Chloramphenicol (30 μg)	57.89 (22/38)	5.26 (2/38)	36.85 (14/38)
Trimethoprim (2.5 μg)	2.60 (1/38)	10.53 (4/38)	86.87 (33/38)

**Table 5:** Minimum inhibitory concentration (MIC) percentages of the four disinfectants against the 14 *Chryseobacterium* species evaluated in this study.

	Minimum Inhibitory Concentration (%)			
Species	Disinfectant 1 (Chloroxylenol)	Disinfectant 2 (Benzalkonium Chloride)	Disinfectant 3 (Chlorhexidine Gluconate)	Disinfectant 4 (Poly-Dimethyl Ammonium Chloride)
*C. balustinum*	0.125	≤ 0.0625	0.25	≤ 0.0625
*C. daecheongense*	0.25	0.25	0.25	≤ 0.0625
*C. formosense*	0.125	≤ 0.0625	0.125	≤ 0.0625
*C. gleum*	0.25	0.25	1.0	≤ 0.0625
*C. indologenes*	0.25	0.125	1.0	≤ 0.0625
*C. indoltheticum*	0.125	≤ 0.0625	≤ 0.0625	≤ 0.0625
*C. joostei*	0.25	0.5	0.5	≤ 0.0625
*C. piscium*	0.125	≤ 0.0625	0.5	≤ 0.0625
*C. scophthalmum*	0.25	≤ 0.0625	0.5	≤ 0.0625
*C. shigense*	0.25	0.125	0.25	≤ 0.0625
*C. soldanellicola*	0.25	≤ 0.0625	0.125	≤ 0.0625
*C. taeanense*	0.125	≤ 0.0625	0.125	≤ 0.0625
*C. taichungense*	0.25	≤ 0.0625	0.125	≤ 0.0625
*C. vrystaatense*	0.25	0.125	0.5	≤ 0.0625

## Data Availability

Data supporting reported results may be requested from the corresponding author.
